# Supplementary material for: Collective radioresistance of T47D breast carcinoma cells is mediated by a Syncytin-1 homologous protein
Source: PLoS One. 2019 Jan 30;14(1):e0206713. doi: 10.1371/journal.pone.0206713 (PMC6353071; doi:10.1371/journal.pone.0206713)
Supplement: S1 File — This file contains supplementary information and experimental data used to prepare Fig 1A, 1B, 1C, Fig 2B and Fig 5A and 5B. (PDF) [file pone.0206713.s001.pdf]

Supplementary Information

Collective radioresistance of T47D breast carcinoma cells is mediated by a Syncytin-1 homologous protein

Roberto Chignola[1,*], Michela Sega[1], Barbara Molesini[1], Anna Baruzzi[2], Sabrina Stella[3], Edoardo Milotti[3]

[1]Department of Biotechnology, University of Verona, Strada Le Grazie 15, 37134, Verona, Italy
[2]Department of Medicine, University of Verona, Piazzale L. Scuro 10, 37134 Verona, Italy
[3]Department of Physics, University of Trieste, Via Valerio 2, 34127 Trieste, Italy

*roberto.chignola@univr.it

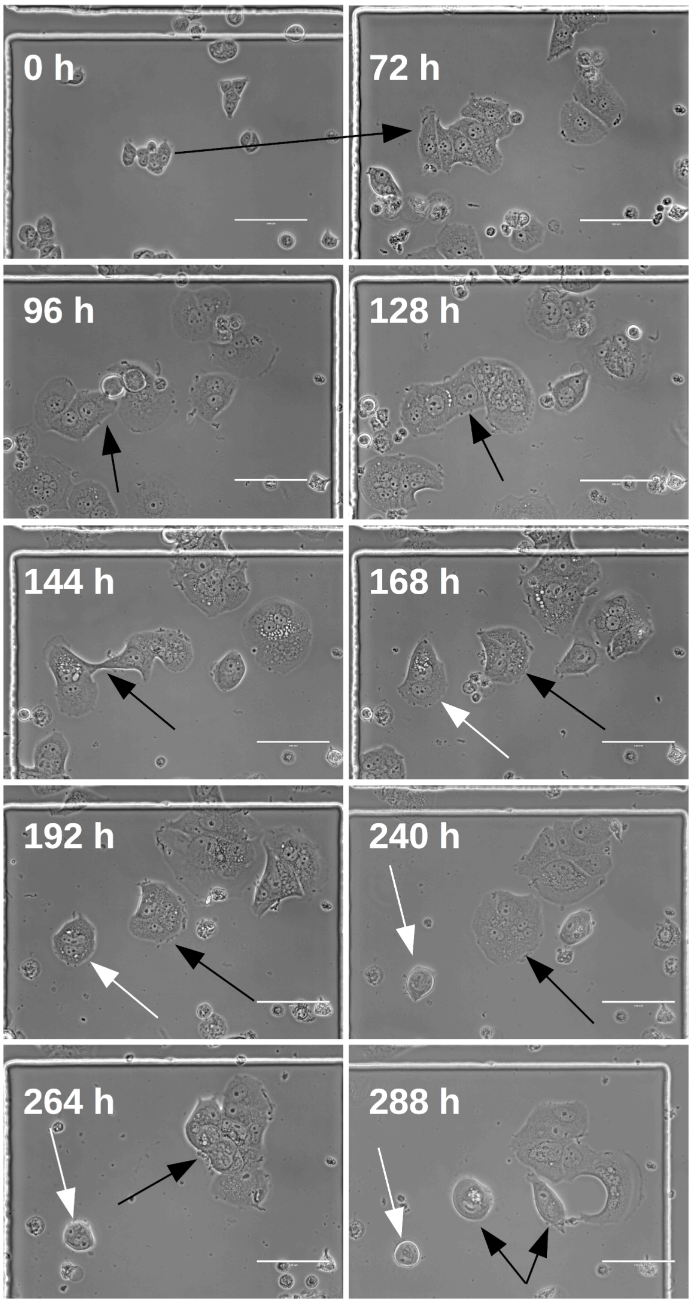

Figure A. Cytological analysis of T47D monolayers after irradiation with 8Gy. Typical examples of microphotographs taken at the indicated post-irradiation times. The microphotograph at 0 hours was taken within 15 min from irradiation. A group of cells is clearly visible. After 72 hours post-irradiation these cells showed an enlarged cell volume (black arrow). At 72 hours some of the individual cell bodies were visible but at later times (96 and 128 hours) cell fusion and the formation of giant cells with multiple nuclei was evident (black arrow). At 144 hours (black arrow), however, a division event took place that produced two giant multinucleated cells. One of these giant cells shrank at later times (white arrows) but the other apparently fused again with neighbour giant cells (black arrows) to form a lump of cells (264 hours, black arrow). Expulsion of cell material from the cluster was then observed after 288 hours post-irradiation. In all panels the reference bar is 100 µm long.

```
CLUSTAL O(1.2.4) multiple sequence alignment


T47Dclone       AGCCCCGCAACAAAAGAGTACCCATTCTTCCTTTTGTTACCGGAGCAGGAGTGCTAGGCA    60
Syncytin-1      AGCCCCGCAACAAAAGAGTACCCATTCTTCCTTTTGTTATAGGAGCAGGAGTGCTAGGTG    60
                ***************************************  *****************

T47Dclone       GACTAGGTACTGGCATTGGTGGTATCACAACCTCTACTCAGTTCTACTATAAACTATCTC    120
Syncytin-1      CACTAGGTACTGGCATTGGCGGTATCACAACCTCTACTCAGTTCTACTACAAACTATCTC    120
                 ****************** ***************************** **********

T47Dclone       AAGAACTAAATGGTGACATGGAATGGGTCGCCAACTCACCAGTCACCTTGCAAGATCAAC    180
Syncytin-1      AAGAACTAAATGGGGACATGGAACGGGTCGCCGACTCCCTGGTCACCTTGCAAGATCAAC    180
                ************* ********* ******** **** *  *******************

T47Dclone       TTAACTTCCTAGCAGCAGTAGTCCTTCAAAATCAAAGAGTTTTAGACTTGCTAACTGCCG    240
Syncytin-1      TTAACTCCCTAGCAGCAGTAGTCCTTCAAAATCGAAGAGCTTTAGACTTGCTAACCGCTG    240
                ****** ************************** ***** *************** ** *

T47Dclone       AAAGAGGGGAAACCTGTTTATTTTTAGGGGAAGAATGCTCTTATTATGTTAATCAATCCG    300
Syncytin-1      AAAGAGGGGGAACCTGTTTATTTTTAGGGGAAGAATGCTGTTATTATGTTAATCAATCCG    300
                ********* ***************************** ********************

T47Dclone       GAATCGTCACCGAGAAAGTTAAAGAAATTCGAGATCGAATACAACGTAGAGCAGAGGAGC    360
Syncytin-1      GAATCGTCACTGAGAAAGTTAAAGAAATTCGAGATCGAATACAACGTAGAGCAGAGGAGC    360
                ********** *************************************************

T47Dclone       TTCAAAACACCAGACCCTGGGACCTCCTCAGCCAATGGGTGTCCTGGATTCTTCCTTTCT    420
Syncytin-1      TTCGAAACACTGGACCCTGGGGCCTCCTCAGCCAATGGATGCCCTGGATTCTCCCCTTCT    420
                *** ******  ********* **************** ** ********** ** ****

T47Dclone       TAGGACCTCTAGCAGTTATAATATTGTTACTTCTCTTTGGACCCTGCATCTTTAACCTGC    480
Syncytin-1      TAGGACCTCTAGCAGCTATAATATTGCTACTCCTCTTTGGACCCTGTATCTTTAACCTCC    480
                *************** ********** **** ************** *********** *

T47Dclone       TTGTTAAGTTTGTCTCTTCCAGAATTGAAGCTGTAAAGCTACAAATGATTCTTCAAATGG    540
Syncytin-1      TTGTTAACTTTGTCTCTTCCAGAATCGAAGCTGTAAAACTACAAAT------------GG    528
                ******* ***************** *********** ********            **

T47Dclone       AGCCCCAGAGGCAGTCCATGACTAAAATCTACTGCGGACCCTTAGACCGGCCTGCCAGCC    600
Syncytin-1      AGCCCAAGATGCAGTCCAAGACTAAGATCTACCGCAGACCCCTGGACCGGCCTGCTAGCC    588
                ***** *** ******** ****** ****** ** ***** * *********** ****

T47Dclone       CATGCTCCAATGTTGATGACATCCAAGGTACCCCTCCGGAGGAAATCTCAACTGCACAAC    660
Syncytin-1      CACGATCTGATGTTAATGACATCAAAGGCACCCCTCCTGAGGAAATCTCAGCTGCACAAC    648
                ** * **  ***** ******** **** ******** ************ *********

T47Dclone       TCCTACTGTGCCCCAATTCAGCAGGAAGCAGTTAGAGCGGTCATCGACAGACCTCCCCAA    720
Syncytin-1      CTCTACTACGCCCCAATTCAGCAGGAAGCAGTTAGAGCGGTCGTCGGCCAACCTCCCCAA    708
                  *****  ********************************* *** *  **********

T47Dclone       CAGCACTTAGGTTTTCCTGATGAGAGGGGGAACTGAGAGACGGGACTAGTTGGATTTCCT    780
Syncytin-1      CAGCACTTAGGTTTTCCTGTTGAGATGGGGGACTGAGAGACAGGACTAGCTGGATTTCCT    768
                ******************* ***** **** ********** ******* **********

T47Dclone       AGGCCAACTAAGAATCCATAAGCCTAGCTGGGAAGGTGACAGCATCCACCTTTAAACACG    840
Syncytin-1      AGGCTGACTAAGAATCCCTAAGCCTAGCTGGGAAGGTGACCACATCCACCTTTAAACACG    828
                ****  *********** **********************  ******************

T47Dclone       GGGCTTGCAACTTAGCTCACACCAGACCAATCAGGTAGTAAAGAGAGCTCACTAAACTGC    900
Syncytin-1      GGGCTTGCAACTTAGCTCACACCTGACCAATCAG---------AGAGCTCACTAAAATGC    879
                *********************** **********         ************* ***

T47Dclone       TAACTAGGCTAAAACAGGAGGTAAAGAAATAGCCAATCATCTATCTCCTGAGAGCACAGC    960
Syncytin-1      TAATTAGGCAAAAACAGGAGGTAAAGAAATAGCCAATCATCTATTGCCTGAGAGCACAGC    939
                *** ***** **********************************  **************

T47Dclone       GGGAGGGACAATGATCGGGA    980
Syncytin-1      AGGAGGGACAATGATCGGGA    959
                 *******************
```

Figure B. Sequence analysis of the 980 bp-long cDNA fragment amplified by RT-PCR in T47D cell extracts. The amplified DNA fragment encompasses a region of 683 nt of the 3’ terminal coding region and a portion 276 nt of the 3’untranslated region of the Syncytin-1 (AF208161) transcript. Alignment between the two DNA sequences was carried out using Clustal Omega software hosted at https://www.ebi.ac.uk.

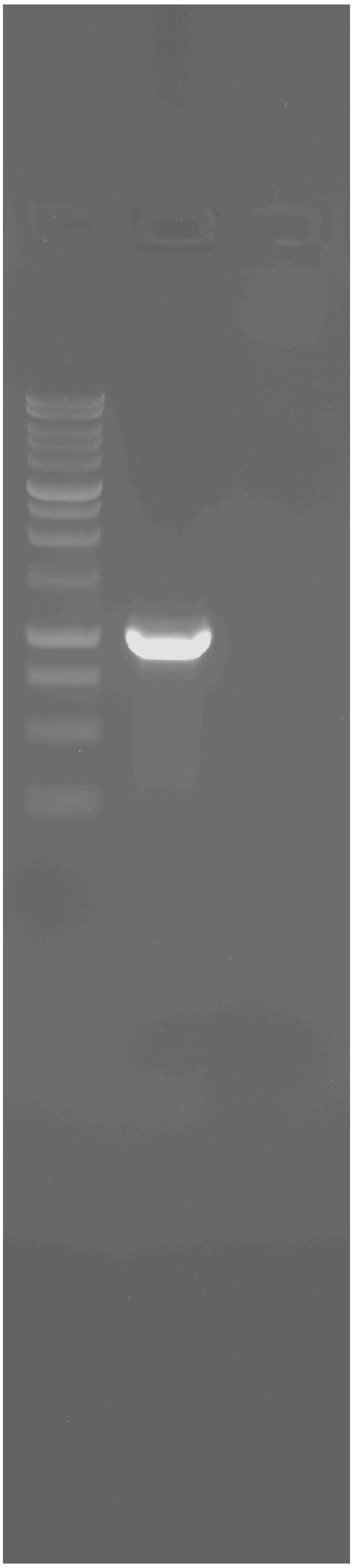

Figure C. Gel elecrophoresis of the DNA fragment amplified by RT-PCR in extracts of T47D cells. Picture of the full-length gel from which we cropped the image reported in Fig.4A in the main text.

Table A. Experimental data shown in Fig.1a, main text. Number of cell colonies surviving radiation treatments

	cells/well											
**Gy**	**100**		**200**		**400**		**800**		**1600**		**3200**	
**0**	25	27	59	48	110	113						
**2**	10	22	24	27	66	76	110	106				
**4**	2	3	9	6	22	16	37	32				
**6**					0	1	3	2	5	5		
**8**									1	1	2	3

Table B. Experimental data shown in Fig.1b, main text. Limiting dilution assays with non irradiated cells: number of wells with no proliferating cells / total number of seeded wells.

	cells/well				
**well geometry**	**1.25**	**2.5**	**5**	**10**	**20**
**F**	53 / 92	48 / 92	21 / 92	10 / 92	1 / 88
**V**	60 / 96	45 / 92	32 / 92	9 / 92	1 / 60

Table C. Experimental data shown in Fig.1c, main text. Number of wells with proliferating cells surviving radiation treatment with 8 Gy. For each condition 96 wells were seeded with the indicated number of cells/well.

		cells/well							
**well geom.**		**39.06**	**78.12**	**156.25**	**312.5**	**625**	**1250**	**2500**	**5000**
**F**	exp.1	3	5	9	9	34	45	68	84
	exp.2	2	3	9	12	39	53	67	85
	mean	2.5	4	9	10.5	36.5	49	67.5	84.5
**V**	exp.1	10	22	31	41	66	78	88	93
	exp.2	11	29	30	55	66	88	90	96
	mean	10.5	25.5	30.5	48	66	83	89	94.5

Table D. Experimental data shown in Fig.2b, main text. Diameters (μm) of cell rings outgrowing sham-irradiated or irradiated spheroids at specific sampling times. Different cell rings (R) are labelled with a progressive number.

	0 Gy					8 Gy				
**Time (hours)**	**R1**	**R2**	**R3**	**R4**	**R5**	**R6**	**R7**	**R8**	**R9**	**R10**
24	120.89	114.83	118.23	122.02	90.55	40.00	72.83	121.51	38.15	107.55
48	227.92	198.44	22061	199.41	164.85	87.02	120.77	173.40	85.94	157.91
120	430.14	363.68	423.89	399.32	351.55	234.30	285.96	344.69	223.01	348.60
144	476.33	428.59	504.61	453.96	417.83	285.48	328.59	377.14	259.68	387.28
168	533.22	489.28	550.24	510.98	476.61	286.81	354.37	394.10	285.16	397.91
192	575.87	523.36	618.09	585.12	541.80	280.27	371.14	400.23	293.57	410.52
216	619.95	567.77	642.82	629.90	583.44	281.96	377.01	398.63	294.59	417.40
288	719.61	669.54	796.83	810.88	746.73	259.58	360.07	347.30	275.76	364.36
312	800.44	732.34	887.47	897.40	820.77	288.84	371.95	360.44	309.78	406.96
336	842.84	775.60	938.16	991.41	884.99	303.10	400.26	345.99	355.78	427.78
360	898.91	811.05	1016.3	1071.3	974.35	323.82	385.81	337.10	361.77	428.17
384	916.77	843.51	1057.9	1122.8	1015.1	287.47	382.31	332.71	347.37	410.52
456	1085.2	940.50	1239.0	1341.3	1211.7	312.35	401.90	365.13	365.80	420.78
504	1174.9	1080.5	1385.7	1494.8	1429.0	316.33	432.10	413.98	360.39	398.23
552	1331.1	1151.2	1508.2	1640.7	1578.8	313.29	438.04	444.92	388.90	458.71
624	1549.3	1283.0	1647.0	1817.6	1700.7	336.21	532.26	545.77	430.59	458.23
672	1591.0	1329.9	1730.4	1980.7	1938.2	341.08	596.84	620.37	455.71	472.45
720	1852.6	1474.1	1889.7	2135.3	2144.9	353.97	659.48	725.15	482.00	532.77
792	2040.0	1567.7	1970.8	2306.7	2376.7	358.59	766.28	847.32	542.85	559.49
888	2391.4	1768.0	2138.8	2480.1	2630.5	357.62	868.53	992.41	638.92	689.54
960	2506.7	1762.3	2088.8	2564.8	2159.1	320.56	949.49	1102.8	717.04	795.10

Table E. Experimental data shown in Fig.5a, main text. Total number of cytoplasmic bridges observed in independent randomly-selected microphotographs (see S4 Fig. below) of cultures of non-irradiated alive cells (A) treated with dead cell bodies (D) from irradiated cultures at different D:A ratios. CT=control samples, i.e. A cells without D dead cell bodies; αHERV=treatment with anti-HERV antibodies.

**CT**		**D:A 1:1**		**D:A 4:1**	
	**αHERV**		**αHERV**		**αHERV**
4	3	17	15	25	19
9	5	20	11	38	16
3	2	21	14	22	21
14	3	18	13	20	15
5	4	17	16	22	21
9	7	22	17	24	19
7	3	11	5	14	20
8	0	12	10	24	6
6	1	16	6	19	15
	0				
	1				
	0				
	3				
	0				
	1				
	0				
	0				
	0				

Table F. Experimental data shown in Fig.5b, main text. Total number of cytoplasmic bridges observed in independent randomly-selected microphotographs of cultures of non-irradiated alive cells (A) treated with: dead cell bodies (D) from irradiated cultures at 1:1 D:A ratio or with cell-free supernatants from irradiated cultures (SN). CT=control samples, i.e. A cells without D dead cell bodies. Cytoplasmic bridges in cell cultures irradiated with a dose of 8 Gy are also given (cells 8Gy).

**CT**	7	6	3	6	9	6	7	7	8	
**D:A 1:1**	13	11	12	13	24	10	8	16	17	7
**SN**	4	7	8	1	4	3	5	1	10	6
**cells 8Gy**	14	20	20	10	16	11	14	11	16	

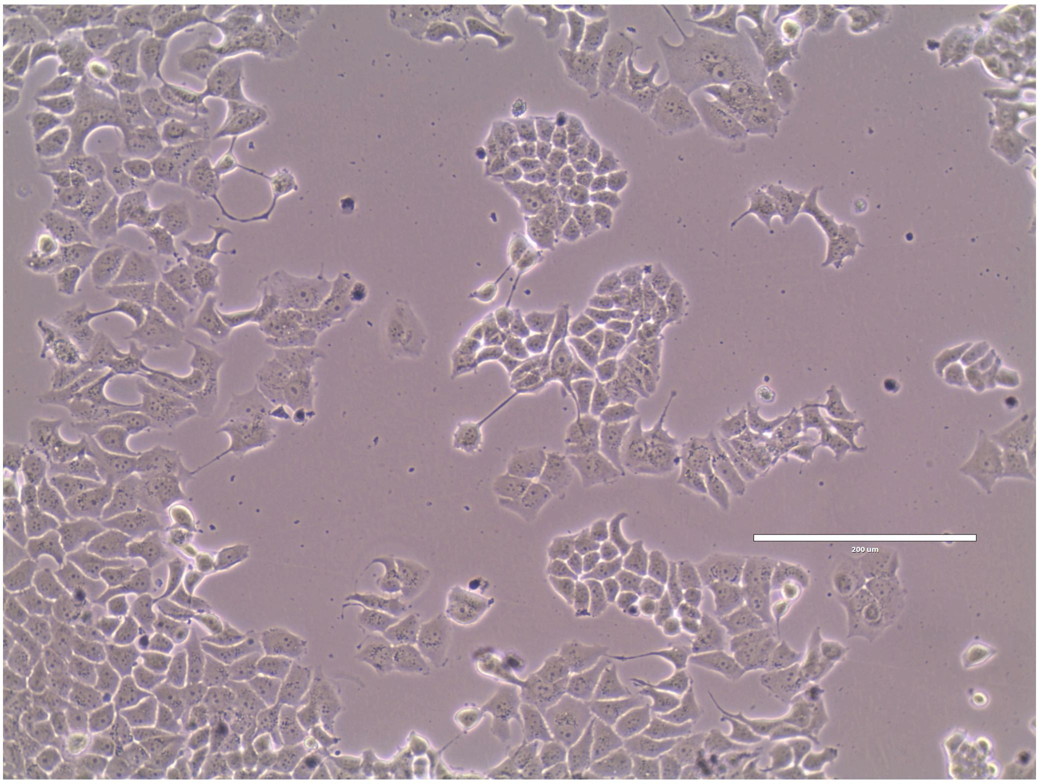

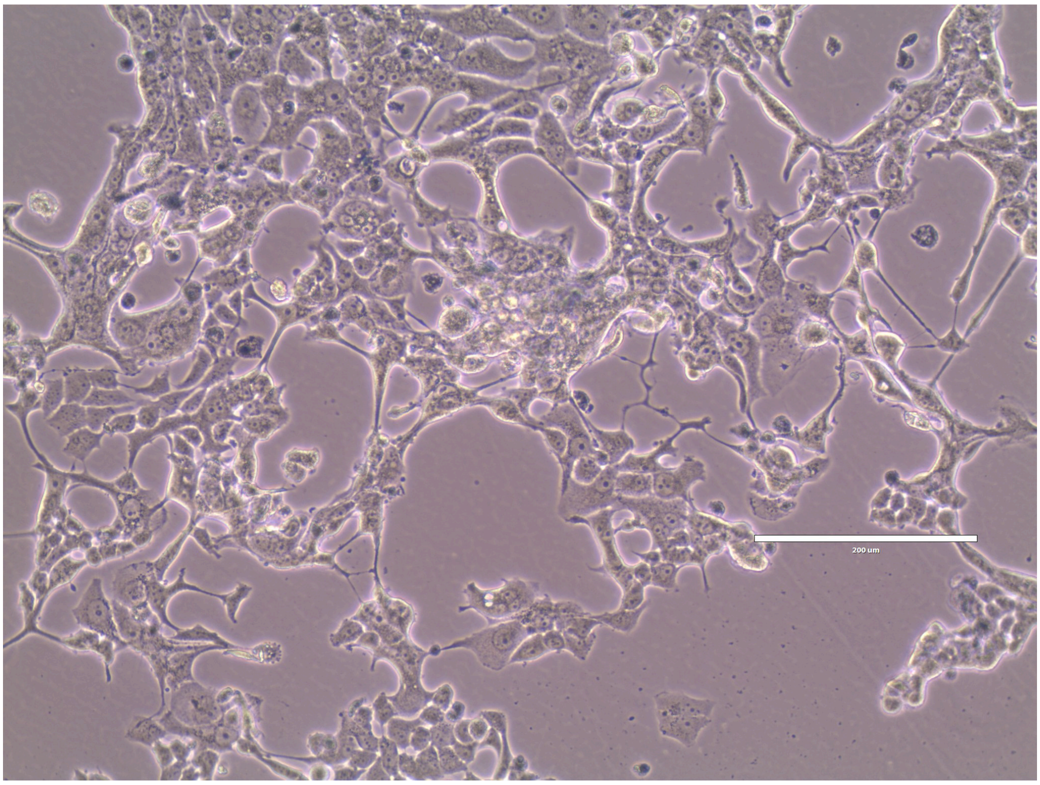

Figure D. Microphotographs from cytoplasmic bridges counting experiments. Two representative examples are shown: top D:A 1:1; bottom D:A 4:1 (see the text in S5 Table). Scale bars are 200 µm long.
